# Prolonged Mechanical Ventilation Induces Cell Cycle Arrest in Newborn Rat Lung

**DOI:** 10.1371/journal.pone.0016910

**Published:** 2011-02-16

**Authors:** Andreas A. Kroon, Jinxia Wang, Brian Kavanagh, Zhen Huang, Maciej Kuliszewski, Johannes B. van Goudoever, Martin Post

**Affiliations:** 1 Physiology and Experimental Medicine Program, Hospital for Sick Children Research Institute, Toronto, Canada; 2 Department of Pediatrics University of Toronto, Toronto, Canada; 3 Department of Physiology, University of Toronto, Toronto, Canada; 4 Departments of Critical Care Medicine and Anesthesia, Hospital for Sick Children, University of Toronto, Toronto, Canada; 5 Department of Pediatrics, Erasmus MC-Sophia, Rotterdam, The Netherlands; 6 Department of Pediatrics, Amsterdam Medical Center Emma's Children's Hospital, Amsterdam, The Netherlands; 7 Department of Pediatrics, Free University Hospital, Amsterdam, The Netherlands; University of Giessen Lung Center, Germany

## Abstract

**Rationale:**

The molecular mechanism(s) by which mechanical ventilation disrupts alveolar development, a hallmark of bronchopulmonary dysplasia, is unknown.

**Objective:**

To determine the effect of 24 h of mechanical ventilation on lung cell cycle regulators, cell proliferation and alveolar formation in newborn rats.

**Methods:**

Seven-day old rats were ventilated with room air for 8, 12 and 24 h using relatively moderate tidal volumes (8.5 mL.kg^−1^).

**Measurement and Main Results:**

Ventilation for 24 h (h) decreased the number of elastin-positive secondary crests and increased the mean linear intercept, indicating arrest of alveolar development. Proliferation (assessed by BrdU incorporation) was halved after 12 h of ventilation and completely arrested after 24 h. Cyclin D1 and E1 mRNA and protein levels were decreased after 8–24 h of ventilation, while that of p27^Kip1^ was significantly increased. Mechanical ventilation for 24 h also increased levels of p57^Kip2^, decreased that of p16^INK4a^, while the levels of p21^Waf/Cip1^ and p15^INK4b^ were unchanged. Increased p27^Kip1^ expression coincided with reduced phosphorylation of p27^Kip1^ at Thr^157^, Thr^187^ and Thr^198^ (p<0.05), thereby promoting its nuclear localization. Similar -but more rapid- changes in cell cycle regulators were noted when 7-day rats were ventilated with high tidal volume (40 mL.kg^−1^) and when fetal lung epithelial cells were subjected to a continuous (17% elongation) cyclic stretch.

**Conclusion:**

This is the first demonstration that prolonged (24 h) of mechanical ventilation causes cell cycle arrest in newborn rat lungs; the arrest occurs in G_1_ and is caused by increased expression and nuclear localization of Cdk inhibitor proteins (p27^Kip1^, p57^Kip2^) from the Kip family.

## Introduction

Introduction of more gentle ventilation strategies -together with surfactant replacement and antenatal corticosteroids- has improved the survival rate of very premature infants. In parallel, the number of infants with ‘new’ bronchopulmonary dysplasia (BPD) [1] has also increased. Currently, infants born at ≤26 weeks of gestation are at the greatest risk of developing such ‘new’ BPD [2], a syndrome of arrested lung development with fewer and larger alveoli and dysmorphic vasculature [3]. BPD can no longer be considered only a pediatric disease because the substantial lung-function abnormalities -and significant symptoms- persist into adulthood [4], [5], [6]. The pathogenesis of BPD is incompletely understood and its treatment is empirical, but mechanical ventilation remains a major risk factor.

Lung development between 24–32 weeks of gestation is characterized by extensive vasculogenesis within the developing terminal saccules, followed by secondary crest formation as well as interstitial extracellular matrix loss and remodeling [7]. This tissue remodeling requires well-coordinated regulation of cell proliferation and apoptosis. Recent studies have shown that prolonged mechanical ventilation increases apoptosis and impairs alveolar septation in newborn mice [8], however the effect of mechanical ventilation on lung cell growth is mostly unknown. *In vitro* studies have demonstrated that mechanical stretch (5% elongation, 60 cycles per min, 15 min/h for 24 h) and oxygen (95%) can inhibit lung cell proliferation [9], [10], but molecular mechanisms are yet to be determined. Cell proliferation is a precisely coordinated set of events regulated by interaction of gene products that activate or suppress cell cycle progression. A series of cyclins and cyclin-dependent kinases (Cdk) act in concert to drive the cycle forward through the G_1_, S and G_2_/M phases [11]. In mammalian cells, G_1_/S transition is an important checkpoint in the cell cycle. Entry into the cell cycle is initiated by mitogen-stimulated expression of D-type cyclins which activate Cdk4/6. Shortly thereafter, cyclin E expression is increased and cyclin E-Cdk2 complexes are formed, promoting entry into the S phase [12]. While cyclin-Cdk complexes positively drive progression of the cell cycle, Cdk inhibitors (CKI) negatively regulate progression by binding to and inactivating cyclin–Cdks [13]. There are two distinct CKI families in mammalian cells: INK4 proteins, which block the progression of the cell cycle by binding to either Cdk4 or Cdk6 and inhibiting the action of cyclin D; and, Cip/Kip proteins that inhibit a broader spectrum of cyclin-Cdk complexes [14], [15], [16].

In this study we determined the effect of prolonged (24 h) mechanical ventilation on lung cell cycle regulators, proliferation and alveolar formation in a newborn rat model [17]. We hypothesized that continuous cyclic (over)stretching of the primitive airsacs would adversely affect proliferation of lung cells by influencing cell cycle regulators.

## Methods

### Ethics statement

The study was conducted according to the guidelines of the Canadian Council for Animal Care and with approval of the Animal Care Review Committee of the Hospital for Sick Children (protocol #7217).

### Animal preparation

Timed pregnant Wistar rats (Charles River, Oakville, Quebec, Canada) were allowed to deliver and immediately afterwards litters were reduced to 10 pups. Newborn rat pups were anesthetized by *i.p*. injection of 30 mg kg^−1^ pentobarbital and a tracheotomy was performed. The trachea was cannulated with a 1 cm 19G cannula and connected to a rodent ventilator (FlexiVent Scireq, Montreal, PQ). Spontaneously breathing, non-ventilated, littermates served as sham controls. One pup per litter was ventilated and a littermate was used as non-ventilated control. Isoflurane was used as general anesthesia during the ventilation period and 0.9% saline (100 ml.kg^−1^/24 h) was administered subcutaneously by continuous infusion with a 27G needle to prevent dehydration. First rat pups at postnatal days 6, 7, 8, 9, 10 and 14 were ventilated to assess lung cell proliferation. For all subsequent experiments 7-day old rat pups were used. Preliminary experiments were performed to determine ventilator settings [18]. Starting from a normal respiratory rate for newborn rats (150 bpm), tidal volume was adjusted to achieve normal blood gas values after the ventilation period. Animals were monitored by ECG. Rectal temperature was maintained at 37°C using a thermal blanket, lamp and plastic wrap. At the end of the ventilation period a blood sample from the carotid artery was taken for blood gas analysis prior to euthanasia.

### Mechanical ventilation

Rat pups were ventilated with room air and moderate-V_T_ (8.5 mL.kg^−1^, RR 150 min^−1^, PEEP 2 cm H_2_O) for 8, 12 and 24 h. In a few cases, pups were ventilated for 4 h with high-V_T_ (40 mL.kg^−1^, RR 30 min^−1^, PEEP 2 cm H_2_O). The 7-day old pups weighed 15.5–18.6 g. Dynamic compliance was estimated every 4 h from data obtained during a single-frequency forced oscillation manoeuvre, using a mathematical model-fitting technique according to the specifications of Scireq Inc. (Montreal, PQ). Two h before completion of ventilation pups were injected *ip* with 50 mg/kg 5-bromo-2-deoxyuridine (BrdU). At completion of ventilation a blood sample was taken from the carotid artery for blood gas analysis and the animals killed by exsanguination. Lung tissues were processed for histology or fresh frozen for molecular/protein analyses.

### Histology

After flushing whole lungs were infused in situ with 4% (w/v) paraformaldehyde (PFA) in PBS with a constant pressure of 20 cm H_2_O over 5 minutes to have equalized filling pressure over the entire lung. Under these constant pressure conditions the cannula was removed and the trachea immediately ligated. The lungs were excised and immersed in 4% PFA in PBS overnight, dehydrated in a ethanol/xylene series and embedded in paraffin. Sections of 5 µm were stained with hematoxilin and eosin or stained for elastin using accustain artrazine solution (Sigma, St. Louis MO, USA).

### Immunohistochemistry

Following sectioning and antigen retrieval by heating in 10 mM sodium citrate pH 6.0, sections were washed in PBS and endogenous peroxidase was blocked in 3% (v/v) H_2_O_2_ in methanol. Blocking was done with 5% (w/v) normal goat serum (NGS) and 1% (w/v) bovine serum albumin (BSA) in PBS. Sections were then incubated overnight at 4°C with either 1∶50 diluted mouse anti-BrdU (Boehringer Mannheim, Germany) or 1∶400 diluted rabbit anti-phospho-histone H3 (Millipore, Billerica, MA, USA) antibodies (Lab Vision Corporation, Fremont, Canada). Biotinylated rabbit anti-mouse IgG or goat anti-rabbit IgG were used as secondary antibodies, respectively. Color detection was performed according to instruction in the Vectastain ABC and DAB kit (Vector Laboratories, Burlingname, CA, USA). All sections were counterstained with hematoxylin. For quantitative analysis, digital images were captured using a Leica digital imaging system at 20× magnification with random sampling of all tissue in an unbiased fashion. Images were captured randomly from 15 non-overlapping fields from each slides, with 3 slides per animal and 4 animals per group.

### Morphometric analysis

Digital images were captured from either H&E- or elastin-stained slides with random sampling of all tissue in an unbiased fashion. Images were captured randomly from 15 non-overlapping fields/slide with 3 slides/animal and 4 animals/group. Tissue fraction was calculated from pixel counts of black/white images [19], mean linear intercepts (Lm) were measured and calculated [20] and the number of elastin-positive secondary septa determined.

### Western blot analysis

Lung tissues were lysed, protein content measured [21] and aliquots (40 g protein) were subjected to 10% SDS-PAGE and transferred to PVDF membranes. After blocking with 5% (w/v) skim milk in TBST (20 mM Tris, 137 mM NaCl, 0.1% Tween 20) membranes were incubated with appropriate primary antibody overnight in 4°C. Because of decreased BrdU incorporation and cyclin D1 and E1 expression, we focused on CKIs inhibiting Cdk-2, -4 and -6 [22]. Primary antibodies were rabbit anti-p15^INK4B^ (dilution of 1∶500), rabbit anti-p16^INK4A^ (dilution of 1∶1000), mouse anti-p21^Waf1/Cip1^ (dilution 1∶500), rabbit anti-p27^Kip1^(dilution 1∶500) and rabbit anti-p57^Kip2^ (dilution of 1∶1000), rabbit anti-cyclin D1 (dilution of 1∶1000) (all from Cell Signaling Technology, Danvers, USA) and rabbit anti-cyclin E1 (dilution of 1∶1000) (Santa Cruz Biotechnology, Santa Cruz USA). Primary phosphorylated p27^Kip1^ antibodies were rabbit anti-p27^Kip1^ (pThr198) (dilution of 1∶400) and rabbit anti-p27^Kip1^ (pSer10)-R (dilution of 1∶2000; both from Santa Cruz Biotechnology, Santa Cruz, USA), rabbit anti-p27^Kip1^ (pThr157) (dilution of 1∶300; R&D Systems Inc, Burlington, Canada) and rabbit anti-p27^Kip1^ (pThr187) (dilution of 1∶400; Novus Biologicals, Littleton, USA). The next day the membranes were washed TBST and incubated with either horseradish peroxidase–conjugated anti-rabbit or anti-mouse IgG (dilution of 1∶1000; Cell Signaling Technology, Danvers, USA). After several washes with TBST, protein bands were visualized using an enhanced chemiluminescence detection kit (Amersham, Piscataway, NJ, USA). Band densities were quantified using Scion Image software (Version 1.6, National Institutes of Health, Bethesda, MD, USA). Equal protein loading was confirmed by immunoblotting for β-actin of same membrane.

### Quantitative RT-PCR

Total RNA was extracted from lung tissues and reverse transcribed [14]. Complementary DNA was amplified for target genes cyclin D1, cyclin E1 and p27 as previously described [17], [19]. For relative quantification, polymerase chain reaction signals were compared between groups after normalization using 18S as internal reference. Fold change was calculated [23].

### Stretch of epithelial cells isolated from fetal rat lungs

Distal fetal lung epithelial cells (day 19 of gestation) were isolated as previously described [24]. Cells were cultured on type-1 collagen-coated BioFlex plates and subjected various durations of cyclic continuous 17% stretch using a FX-4000 Flexercell Strain Unit (Flexercell Int., NC, USA) [25]. Neither cell viability (trypan blue exclusion) nor cell attachment was affected by the duration of applied stretch regimen. Cell lysates were processed for Western Blotting.

### Statistical analysis

Stated otherwise all data are presented as mean ± SD. Data was analyzed using SPSS software version 15 (SPSS Inc, Chicago, IL). Statistical significance (p<0.05) was determined by using one-way ANOVA or Kruskal-Wallis test. *Post hoc* analysis was performed using Duncan's multiple-range test (data presented as mean ± SD) or Mann-Whitney test (data presented as median and interquartile range).

## Results

### Physiologic data

**Figure 1:**
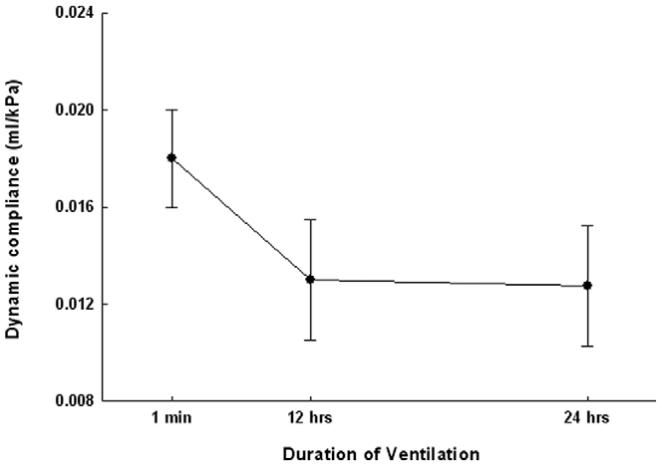
Dynamic compliance during 24 h of mechanical ventilation. Dynamic compliance decreased during first 12 h of ventilation with room air and low/moderate V_T_ but remained stable during the next 12 h. Data are mean ± SD, n = 12 rat pups per time group. *p<0.05 prolonged versus 1-min ventilation.

Blood gases were in the normal range after 8, 12 and 24 h of ventilation (Table 1). Mean airway pressures, peak pressures and delivered V_T_ remained constant up to 8 h of ventilation [18], but altered slightly after 12 h of ventilation compared to baseline (Table 1). Dynamic compliance of the respiratory system was constant up to 8 h of ventilation [18] decreased after 12 h and remained stable afterwards (Fig. 1). These results are indicative of the impact of 8 h of ventilation that did not subsequently worsen.

**Table 1:** Blood gas analysis and airway pressures after 8, 12 and 24 h of ventilation.

	8 hrs	12 hrs	24 hrs
pH	7.39±0.07	7.29±0.05	7.30±0.05
pCO_2_ (mmHg)	44.3±6.4	35.8±6.2	39.4±4.5
pO_2_ (mmHg)	83±8.4	87.4±11.2	73.5±11.7
BE (mmol/L)	−4.2±2.3	−3.9±1.6	−5.2±2.1
Ppeak (cm H_2_O)	10.9±1.1	12.7±1.1*	13.1±1.3*
Pmean (cm H_2_O)	6.3±0.5	7.2±0.5*	7.5±0.6*
PEEP (cm H_2_O)	2	2	2
Frequency (breaths/min)	150	150	150
Delivered V_T_ (ml/kg)	8.9±0.2	8.5±0.4*	8.4±0.2*

Values represent means ± SD, n = 12 animals per group.

*p<0.05 versus values at 0 hrs. Ppeak, peak pressure; Pmean, mean pressure; PEEP, positive-end expiratory pressure.

### Morphometric analyses

**Figure 2:**
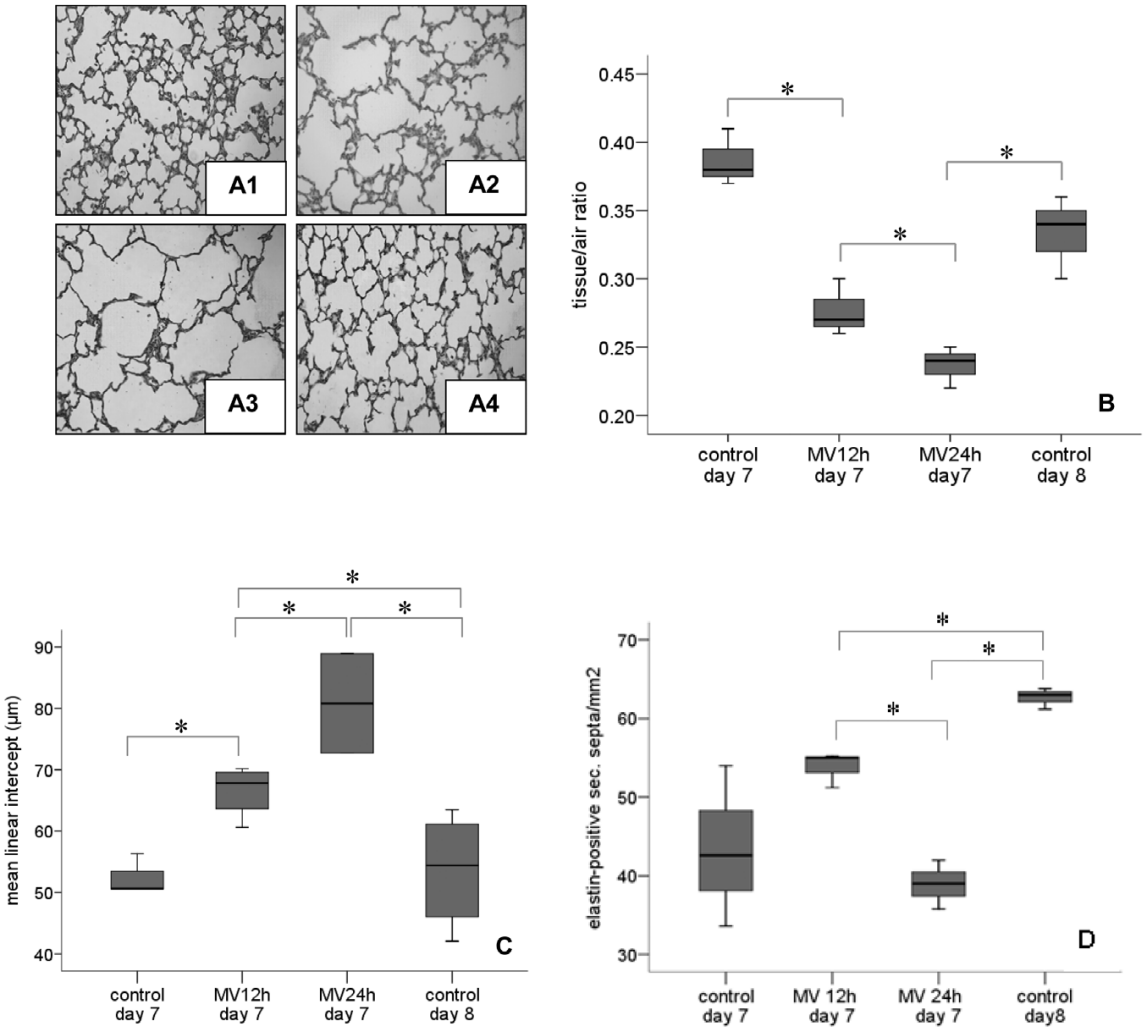
Ventilation inhibits alveolar growth. (A) Histology after mechanical ventilation: (A1) non-ventilated 7-day old rat (A2) 7-day old rat ventilated for 12 h, (A3) 7-day old rat ventilated for 24 h, (A4) non-ventilated 8-day old rat. (B) Mechanical ventilation for 12 and 24 h significantly increased alveolar airspace (reduction in tissue-to-air ratio (A) as well as increase in mean linear intercept (D)) but decreased number of elastin-positive secondary septa (C). Medians with 25^th^ and 75^th^ quartiles are shown, bars are 5^th^ and 95^th^ percentiles, n = 12 rat pups per time group. MV, mechanical ventilation. * p<0.05.

Seven-day old rat pups ventilated for 12 and 24 h had fewer and larger alveoli when compared to the lungs of non-ventilated 8 day-old pups (Fig. 2A). The tissue-to-air ratio corroborated these findings; it decreased after 12 h of ventilation and declined further during the next 12 h of ventilation (Fig. 2B). To quantify alveolar development, we calculated the number of elastin-positive secondary crests per unit area (Fig. 2D). The number of secondary crests -indicating alveolar formation- increased significantly between the 7^th^ and 8^th^ postnatal days in non-ventilated rat pups. The number of secondary crests increased after 12 h of ventilation when compared to day 7 controls. In contrast, the number of secondary crests was significantly lower in pups ventilated for 24 h *vs*. non-ventilated day 8 control pups, even when corrected for tissue fraction. To further evaluate alveolar development, we measured the mean linear intercept (Lm; Fig. 2C). Ventilation increased the Lm after 12 h, and more so after 24 h.

Together the data suggest that during the first 12 h of ventilation alveolar space increases because of hyperinflation while a further increase of alveolar space during the next 12 h of ventilation is in part due to arrest in alveolar development as well as hyperinflation.

### Lung cell proliferation

**Figure 3:**
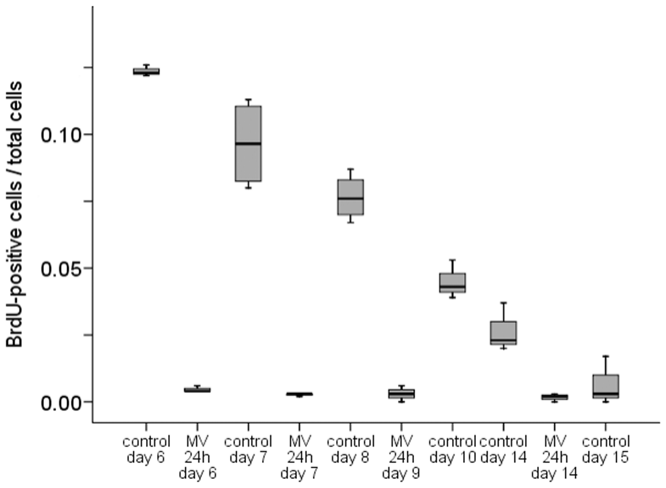
Ventilation inhibits BrdU uptake independent of postnatal age. Although the BrdU labeling index decreased gradually with advancing postnatal gestation, mechanical ventilation for 24 h inhibited cell proliferation at every postnatal age. Medians with 25^th^ and 75^th^ quartiles are shown, bars are 5^th^ and 95^th^ percentiles, n = 4 rat pups per time group. *p<0.05.

**Figure 4:**
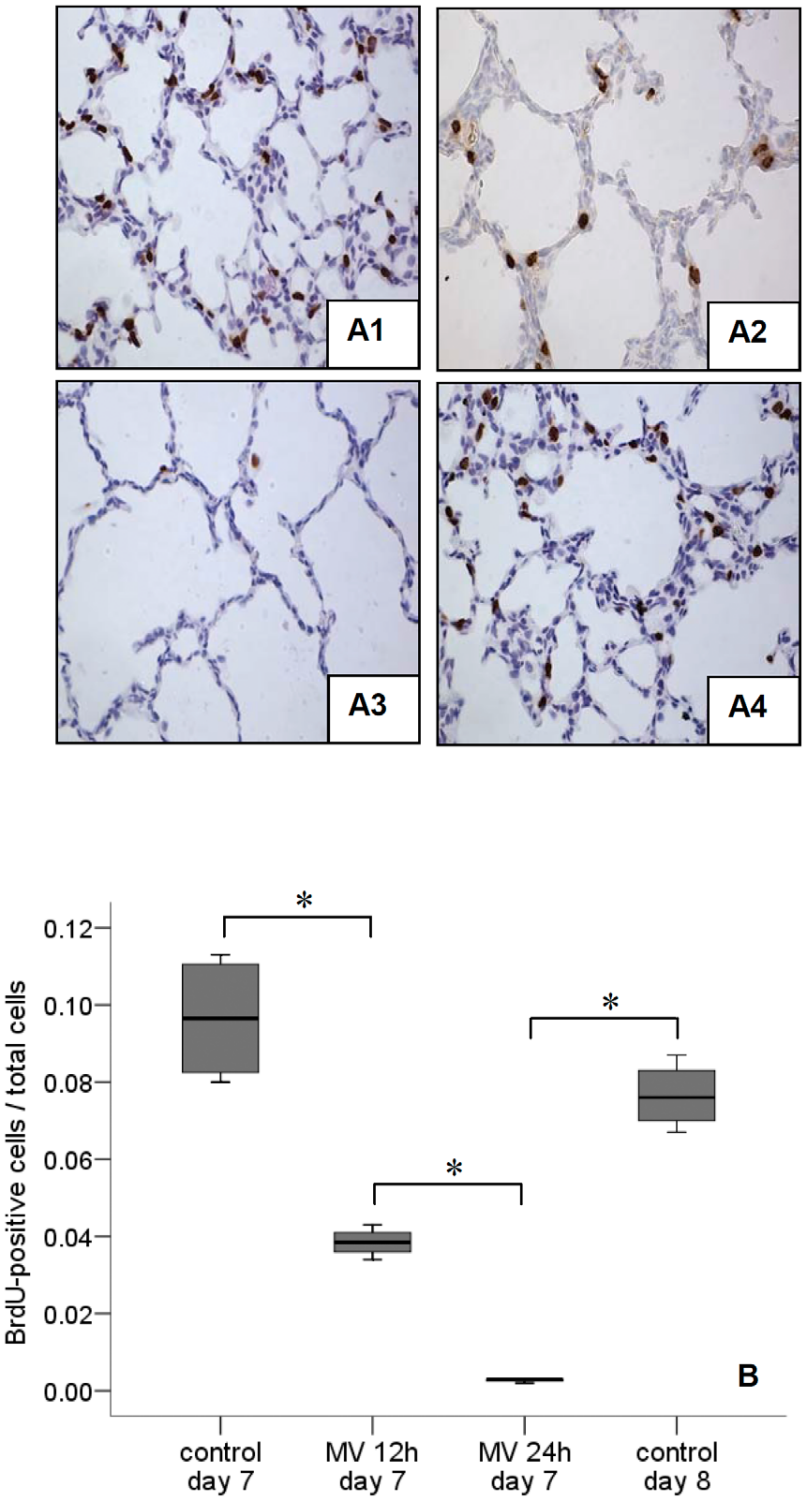
Ventilation inhibits lung cell growth. Immunohistochemistry ((A1) non-ventilated 7-day old rat, (A2) 7-day old rat ventilated for 12 h, (A3) 7-day old rat ventilated for 24 h, (A4) non-ventilated 8-day old rat) illustrates reduction in BrdU uptake (brown color) with duration of ventilation. (B) BrdU labeling index significantly decreased after 12 and 24 h of mechanical ventilation. Medians with 25^th^ and 75^th^ quartiles are shown, bars are 5^th^ and 95^th^ percentiles, n = 12 rat pups per time group. MV, mechanical ventilation. * p<0.05.

Lung cell proliferation was assessed in non-ventilated *vs*. ventilated rat pups at postnatal days 6, 7, 8, 9, 10 and 14. In non-ventilated rats, the number of proliferating lung cells was greatest at postnatal day 6 (BrdU labelling index: ∼12%), which declined gradually to almost undetectable at day 15 (Fig. 3). Ventilation for 24 h clearly inhibited lung cell proliferation in pups of all studied ages (days 6-14). Next, 7-day old rat pups were ventilated for all subsequent experiments. Proliferation was not affected by 8 h of ventilation (data not shown) but longer durations of ventilation significantly decreased the number of proliferating cells (Fig. 4A, B). The ratio of proliferating mesenchymal and epithelial cells did not significantly differ between non-ventilated pups and pups ventilated for 8 and 12 h, respectively (0.73±0.05 vs. 0.65±0.03 and 0.67±0.1). Since a 12-h ventilation decreased the total number of proliferating cells (Fig. 4B) the unchanged ratio suggest that cell proliferation of both tissue layers was equally affected by mechanical ventilation. Hardly any proliferating cells were seen after 24 h of ventilation; in agreement with a reduction in cell proliferation in both tissue layers. The almost total arrest in lung cell proliferation by prolonged (24 h) ventilation was confirmed by anti-PH3 immunochemistry (PH3-positive cells decreased from 8 to 1% of total).

### Cell cycle regulators

**Figure 5:**
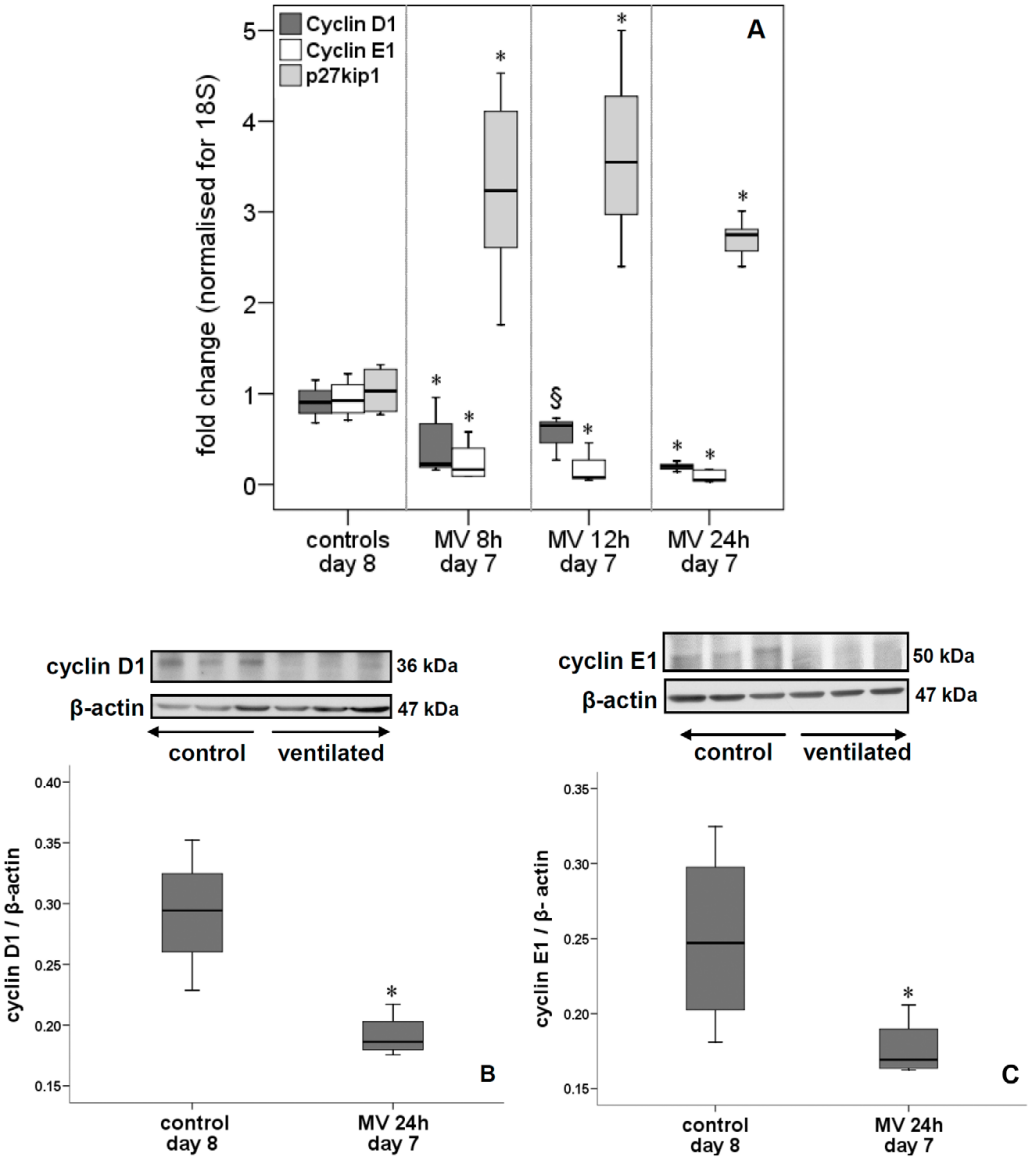
Impact on cyclin D, cyclin E and p27^Kip1^ expression. Mechanical ventilation for 24 h significantly decreased cyclin D1 and E1 mRNA (A) and protein (B and C) levels in lungs of 7-day old rats. In contrast, p27^ kip1^ mRNA increased (A). Inserts in (B) and (C) show cyclin D1 (B) and cyclin E1 (C) immunoblots of lung tissue of 7-day old rats ventilated for 24 h and non-ventilated 8-day old rats (controls). Blots were reprobed with β-actin for equal loading and transfer. Medians with 25^th^ and 75^th^ quartiles are shown, bars are 5^th^ and 95^th^ percentiles; qPCR, n = 6 rat pups per time group; immunoblot, n = 3 rat pups per time group. MV, mechanical ventilation. *p<0.05 versus non-ventilated group, ^§^ p<0.05 versus 24 h ventilation.

**Figure 6:**
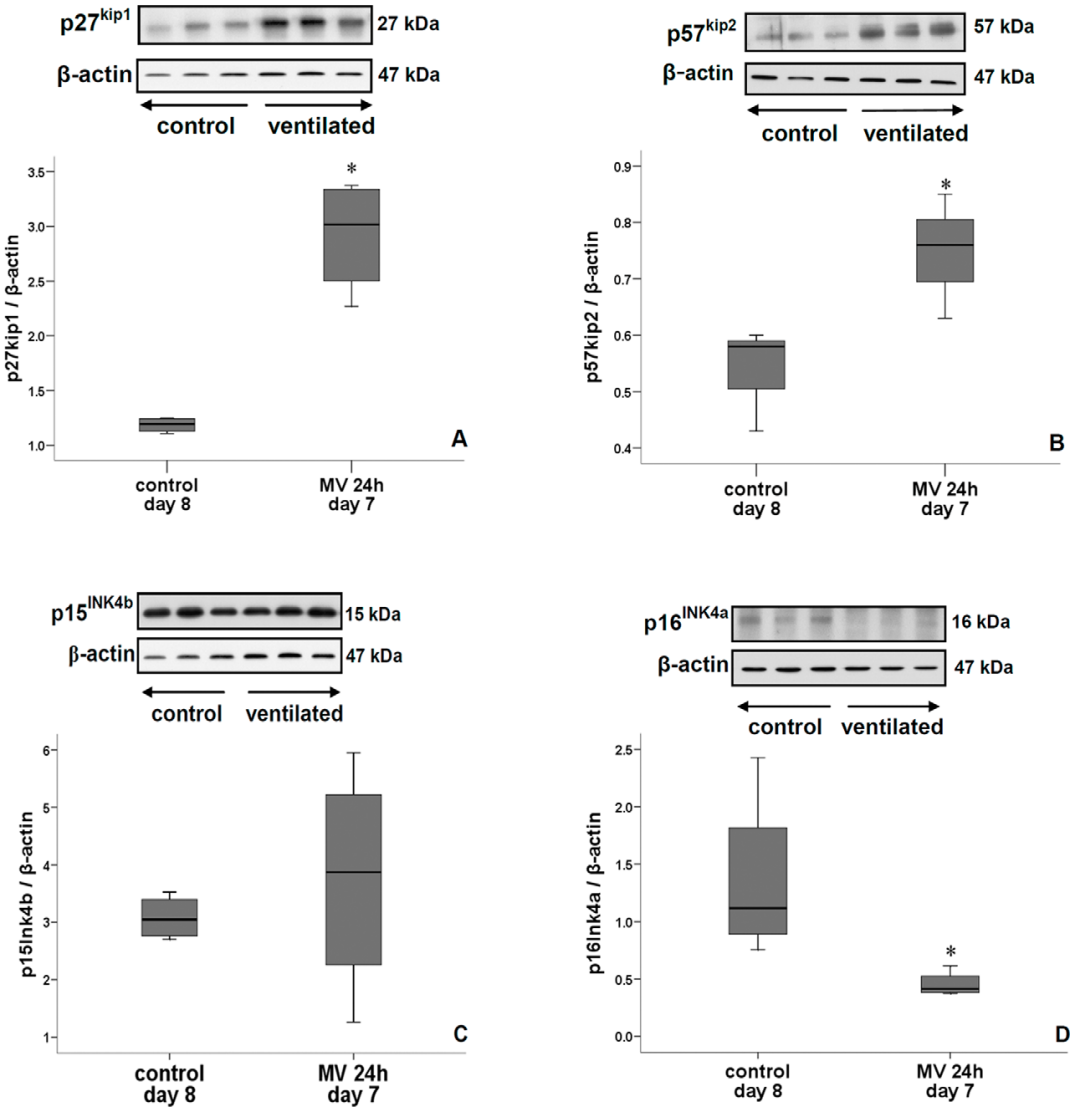
Different effects on Kip and INK proteins. Mechanical ventilation for 24 h significantly increased p27^ Kip1^ (A) and p57^ Kip2^ (B) protein levels. In contrast, p16^ INK4a^ protein (D) was decreased by ventilation while p15^ INK4b^ (C) was unchanged. Inserts show immunoblots of lung tissue of 7-day old rats ventilated for 24 h and non-ventilated 8-day old rats (controls). Blots were reprobed with β-actin for equal loading and transfer. Medians with 25^th^ and 75^th^ quartiles are shown, bars are 5^th^ and 95^th^ percentiles; n = 3 rat pups per time group. *p<0.05 versus non-ventilated group.

**Figure 7:**
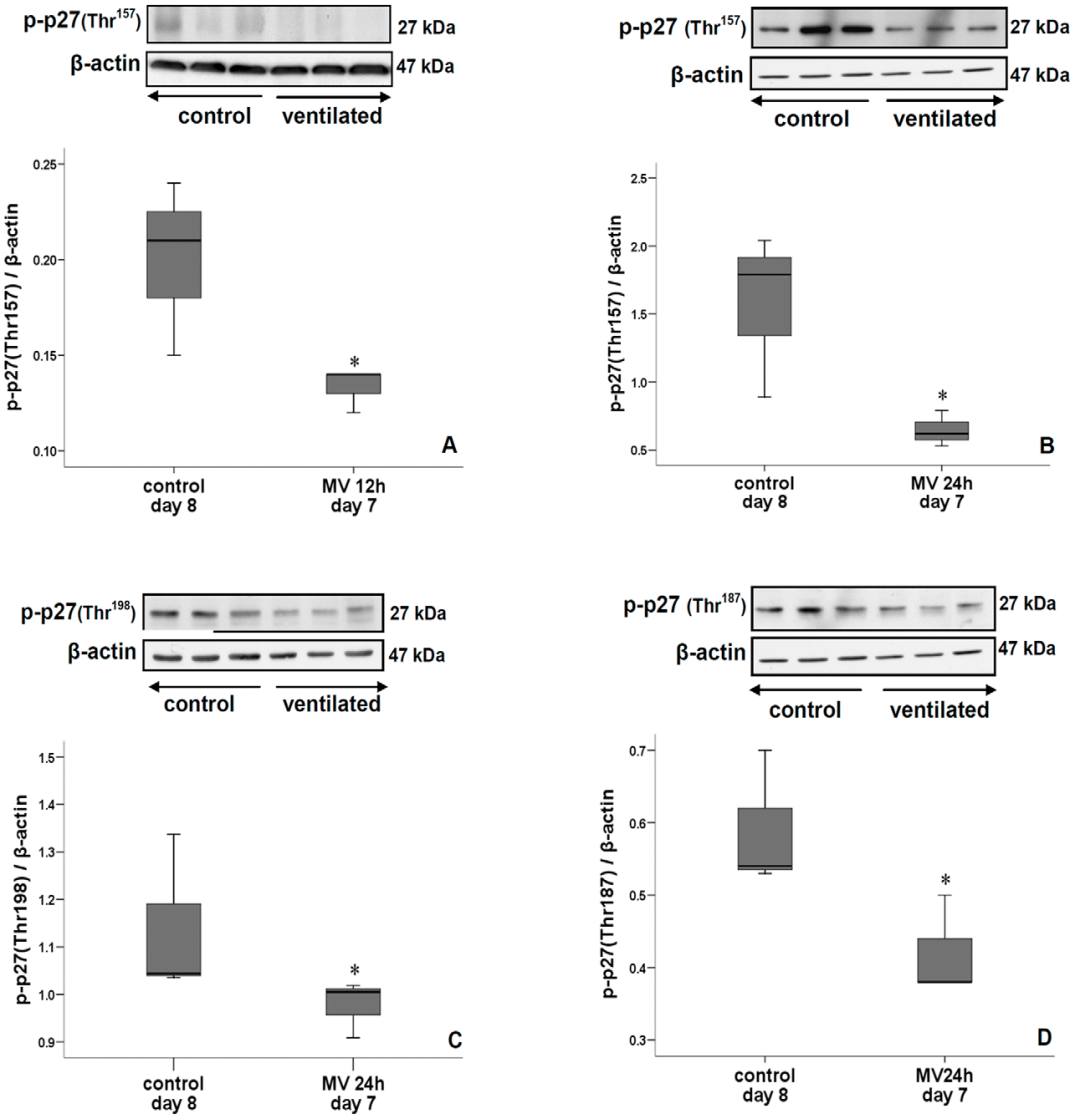
Effect on p27^ kip1^ phosphorylation. A 24 h-ventilation significantly decreased Thr^157^-phosphorylated p27^Kip1^ (A), Thr^187^-phosphorylated p27^Kip1^ (B) and Thr^198^-phosphorylated p27^Kip1^ (C). Phosphorylation of threonine 157 was already reduced after 12 h of ventilation (D). Inserts show immunoblots of lung tissue of 7-day old rats ventilated for 24 h and non-ventilated 8-day old rats (controls Blots were reprobed with β-actin for equal loading and transfer. Medians with 25^th^ and 75^th^ quartiles are shown, bars are 5^th^ and 95^th^ percentiles; n = 3 rat pups per time group. *p<0.05 versus non-ventilated group.

mRNA levels of lung cyclin D1 and E1 were significantly down-regulated after 8, 12 and 24 h of ventilation while that of p27^Kip1^ was increased (Fig. 5A). Immunoblot (i.e. protein) analysis of lungs ventilated for 24 h confirmed these mRNA changes of cyclin D1, E1 and p27^Kip1^ (Figs. 5B, 5C, 6A). The amount of p27^Kip1^ was 1.5-fold increased after 12 h of ventilation (not shown). Other members of the Cip/Kip family of CKIs were either increased (p57^Kip2^, Fig. 6B) or unchanged (p21^Waf/Cip1^, not shown) by 24 h of ventilation. In contrast, CKIs belonging to the INK4 family were either reduced (p16^INK4a^) or not affected (p15^INK4b^) by 24 h of ventilation (Fig. 6C, D). p27^Kip1^ can be phosphorylated at different sites, which influences its localization and activity [26]. A 12 h ventilation decreased phosphorylation of p27^Kip1^at Thr^157^ (Fig. 7A) but did not affect phosphorylation of Thr^198^ (not shown). However, mechanical ventilation for 24 h decreased p27^Kip1^ phosphorylation at Thr^157^, Thr^187^ and Thr^198^, thereby promoting stability and nuclear localization (Fig. 7B–D). Similar -but more rapid- changes in cell cycle regulators were noted when 7-day newborn rats were ventilated with high V_T_. Although β-actin can be responsive to stretch, no significant differences in β-actin expression was noted between ventilated animals and controls (not shown).

**Figure 8:**
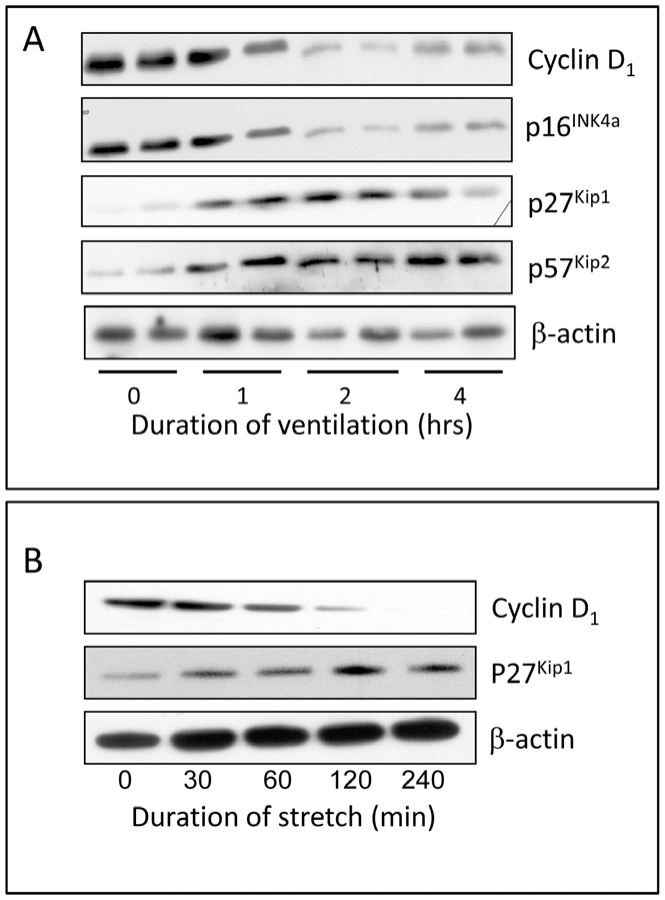
Effect of high tidal volume ventilation and in vitro stretch. High V_T_ ventilation of 7-day old rat lungs (A) and a continuous cyclic 17% stretch of fetal lung epithelial cells (B) rapidly decreased type-D cyclins and p^16INK4a^ while increasing Kip proteins, in particular p27^Kip1^. Blots were reprobed with β-actin for equal loading and transfer. Representative blots of 2 experiments carried out in duplicate (A) or 3 experiments (B).

High V_T_ reduced the amount of D1 and D2 cyclins within 1 hour, while that of Cdk inhibitors p27^Kip1^ and p57^Kip2^ increased (Fig. 8A); in contrast, p16^INK4a^ content was decreased by high-V_T_ ventilation.

We do not know in which particular tissue layer (epithelium, mesenchyme) these changes occurred *in vivo*, but they at least occur in epithelial cells as subjecting *ex vivo* fetal lung epithelial cells to cyclic continuous 17% stretch resulted in similar patterns of alteration in cell cycle regulators (Fig. 8B).

## Discussion

The hallmark of ‘new’ BPD is arrested alveolarization [1], but the molecular and cellular basis of the alveolar arrest remains mostly unknown. Alveolarization occurs as the immature saccules, which form the lung parenchyma at birth, are subdivided into smaller units by the formation and extension of secondary septa; new tissue ridges are lifted off the existing primary septa and grow in a centripetal direction into the airspaces. This process, called septation, is mainly postnatal (human: 36 weeks-infancy; rat: Pnd5–Pnd21) [7], [27]. Before septation of the air spaces starts, the lung expands for a short period of time, and the cells of the inter-airway walls actively proliferate, peaking at day 5 in rats and steadily declining thereafter [28]. This active cell proliferation takes place just at the beginning of the septation of the distal airways. With the use of a newborn rat model [18] we demonstrate here that mechanical ventilation for 24 h with room air and moderate V_T_ results in cell cycle arrest, and reduced alveolar septation. This ventilation strategy (room air and moderate VT) was chosen to avoid/minimize lung injury.

In rats, lungs at birth have a saccular appearance and alveolarization is an exclusively postnatal (between P4 and P21) event, which makes this model relevant to the infant population developing BPD. However, major differences exist between mechanically ventilated newborn rats and premature born infants. Newborn rats have immature lung architecture at birth, but they do not need mechanical ventilation to survive (likely due to differences in airway structure, with large airways extending almost to the lung periphery and quickly reducing in diameter to the alveoli) and they do not lack surfactant. Infants with BPD demonstrate interstitial thickening that may partly be due to fibroproliferation while in rat pups mechanical ventilation of 24 h caused cell arrest in both mesenchymal and epithelial cell layers. Despite these differences, our results suggest that the observed cell cycle arrest is due to increased expression of two CKIs (i.e. p27^Kip1^ and p57^Kip2^) that are members of the Cip/Kip family; the other member, p21^waf/Cip1^, was not affected by 24 h of mechanical ventilation.

Knock-in mouse models have shown that p27^Kip1^ and p57^Kip2^ are interchangeable *in vivo* [29], suggesting similar mechanisms of regulation. Mechanical strain has been recognized as playing an important role in the regulation of fetal lung cell proliferation. Indeed the stimulatory effect of mechanical stretch (i.e. increased intratracheal pressure) on fetal lung growth has been extensively studied in tracheal occlusion (TO) models [30], [31], where the number of proliferating alveolar type II cells significantly increased. Fetal sheep, exposed to TO during the alveolar stage of lung development, showed an increase in alveolar type II cells between days 2-4 after TO [31]. This proliferative response of fetal lung cells to strain has also been demonstrated *in vitro.* Intermittent cyclic 5% stretching (simulating normal fetal breathing movements) of distal fetal rat lung cells (epithelial cells and fibroblasts) increased cell proliferation [32]. However, a continuous cyclic 17% stretch (simulating mechanical ventilation [33]) for 24 h inhibited fetal lung cell proliferation (unpublished results), in agreement with our *in vivo* findings of a proliferative arrest after 24 h of mechanical ventilation. In the present study, continuous cyclic 17% stretch of fetal lung epithelial cells caused similar alterations in cell cycle regulators as observed in mechanically ventilated newborn lungs *in vivo*, namely increased levels of p27^Kip1^ and a decrease in the amount of cyclin D1. CKI members of the Cip/Kip family (p21^WAF1/Cip1^, p27^Kip1^ and p57^Kip2^) preferentially inhibit cyclin-Cdk2 complexes [16]. How mechanical stretch influences CKIs is unknown. In many cancers, the ras/raf/mitogen activated protein kinase (MAPK) pathway increases p27^Kip1^ proteolysis while downstream effectors of the PI-3K pathway such as protein kinase B (also known as Akt) predominantly regulate p27^Kip1^ subcellular localization [26]. Although the MAPK pathway is activated by ventilation/stretch [34], [35] we found nuclear p27^Kip1^ accumulation instead of degradation. Thus, MAPK may regulate p27^Kip1^ differently in normal compared to cancer cells. The PI3K-Akt pathway during ventilation/stretch remains to be investigated. Mechanical ventilation of newborn rats triggers an inflammatory response[17], [18] and various inflammatory mediators including tumor necrosis factor-a (TNFα, interleukin-6 and transforming growth factor-β (TGF-β) have been shown to induce p21^WAF1/Cip1^ expression [22], [36], [37]. Also p15^Ink4b^ is induced by TGF-β [38]. In the current study, TGFβ_1_ mRNA expression was not changed after 24 h of ventilation (data not shown) and, indeed, neither p21^WAF1/Cip1^ nor p15^Ink4b^ expression was affected by mechanical ventilation.

The amount of p27^Kip1^ is regulated at the level of its synthesis (transcription, translation), degradation and localization [39]. During the G_0_ phase, it accumulates in the nucleus and inhibits cyclin-Cdk complexes. In response to growth stimuli, p27^Kip1^ translocates from the nucleus to the cytoplasm during G_1_ phase and is degraded by the proteosome after ubiquitination [39], permitting the cell cycle to progress to S phase. Several signaling pathways that alter p27^Kip1^ phosphorylation influence its subcellular localization and function. For example, phosphorylation of the following essential sites regulate important functions: Thr^157^ prevents nuclear import; Ser^10^ mediates nuclear export; Thr^198^ promotes cytoplasmic translocation and increases p27-dependent motility, which may be important to prepare cells for shape changes in later phases of the cell cycle; and, Thr^187^ results in proteolysis [26], [39]. In the present study, mechanical ventilation of 24 h increased the transcription of p27^Kip1^ and altered its phosphorylation: less phosphorylation of Thr^157^ (increasing nuclear import), Thr^198^ (decreasing nuclear export) and Thr^187^ (reduced proteolysis). No significant changes in Ser^10^ phosphorylation were noted (not shown). Together, these alterations in p27^Kip1^ phosphorylation favour its nuclear localization and stability. In addition, the reduced phosphorylation of p27^Kip1^ at Thr^157^ and Thr^198^ negatively affects the assembly function of p27^Kip1^ for cyclinD1-Cdk4, thereby negatively affecting cell cycle progression [26].

The second family of CKIs are INK4 proteins (p16^INK4a^, p15^INK4b^, p18^INK4c^ and p19^INK4d^); they inhibit cyclin D-dependent kinases Cdk-4 and -6 [14], [15] and, are thus specific for early G_1_ phase. In the present study, we found a significant reduction in p16^INK4a^ protein after ventilation with low, moderate or high V_T_. In addition to Cdk inhibition and G_1_ growth arrest, p16^INK4a^ plays a role in regulating apoptosis. It has been shown that p16^INK4a^-deficiency increases apoptosis in osteosarcoma U2OS and mouse embryonic fibroblast (MEF) cells exposed to ultraviolet (UV) light [40], because of down-regulation of the anti-apoptotic protein Bcl-2. In contrast, the pro-apoptotic protein Bax was down-regulated in p16^INK4a^ expressing cells [40]. Thus, p16^INK4a^ appears to control apoptosis through the intrinsic mitochondrial death pathway. Prolonged mechanical ventilation has been shown to significantly increase lung cell apoptosis in newborn mice lungs [8]. Although p16^INK4a^ levels were decreased in the present study, it remains yet to be determined whether it plays a role in ventilator-induced apoptosis.

Another risk factor for BPD is oxygen [1]. Hyperoxia has been shown to interfere with cell-cycle progression *in vitro* [36], [41], [42] and hyperoxia-induced G_1_ arrest appears to be mediated by p21^Waf1/Cip1^ [43], [44]. The hyperoxic induction of p21^Waf1/Cip1^ is p53-dependent [44] and its increase promotes survival of cells exposed to continuous oxidative stress by maintaining anti-apoptotic Bcl-2X(L) expression [45]. Hyperoxic-ventilated premature baboons delivered at 125 and 140 days of gestation have increased p53 and p21^Waf1/Cip1^ expression [46], [47]. In the present study, we did not assess p53 but the absence of p21^Waf1/Cip1^ induction by 24 h of mechanical ventilation with room air suggests that p53 is likely not involved in ventilation-induced cell cycle arrest in these studies.

The increase of p27^Kip1^ and p57^Kip2^ by mechanical stretch *in vitro* and *in vivo* coincided with a reduced expression of cyclins D1 and E1, both of which are essential for cell cycle progression through G_1_ and entry in S phase. D-type cyclins are induced by mitogenic stimuli in quiescent cells. After association with Cdk4/6 and activation by Cdk activating kinase, they promote entry into the G_1_ phase, thereby triggering cyclin E expression. Cyclin E binds to Cdk2 and facilitates transition from G_1_ to S phase [22]. Both p27^Kip1^ and p57^Kip2^ are potent inhibitors of cyclin E-dependent kinase Cdk2, but at high concentrations they also block Cdks4/6. In addition, it is plausible that cell cycle progression is inhibited due to the reduced phosphorylation of p27^Kip1^ at critical threonines (Thr157, Thr198) which negatively affects the assembly function of p27^Kip1^ for cyclin D1-Cdk4 complexes [48]. The down-regulation of cyclin D1 and E1 expression suggests a G_1_ cell cycle arrest, a conclusion that is supported by the absence of BrdU incorporation (S-phase event) and positive PH3 staining (M-phase marker). In the 125-day premature born baboon model of BPD, the animals received ventilator support and oxygen as needed to achieve normal blood-gas values [49], and such treatment increased pulmonary expression of cyclin D1 and E at day 6 while prolonged ventilation and oxygen exposure led to a decrease in cyclin E [46]. It is possible that lung cells were initially undergoing repair by increasing proliferation, but that prolonged exposure impairs the expression of cyclins, resulting in failure of repair and inhibition of further development. Furthermore, increased levels of the Cdk inhibitor p21^Waf1/Cip1^ in the baboon BPD model [46] suggests that G_1_ growth arrest may occur in infants with BPD. Unfortunately, the expression of p27^Kip1^ or p57^Kip2^ has not been investigated in the baboon BPD model.

In summary, we conclude that mechanical ventilation for 24 h using moderate V_T_ without supplemental O_2_ causes G_1_ cell cycle arrest of lung cells in newborn rats due to increased transcription and altered phosphorylation (in favour of nuclear localization) of Kip CKIs, and down-regulation of cyclins D and E. This proliferative arrest may cause a reduction in alveolarization, resulting in alveolar simplification. Such identification of ventilation-induced CKIs may have therapeutic potential for the prevention -or treatment- of arrested alveolarization in ventilated premature infants.
