# Correction: Prolonged Mechanical Ventilation Induces Cell Cycle Arrest in Newborn Rat Lung

**DOI:** 10.1371/annotation/23c9512d-c429-4673-a640-35cc2e4c2c5b

**Published:** 2011-03-21

**Authors:** Andreas A. Kroon, Jinxia Wang, Brian Kavanagh, Zhen Huang, Maciej Kuliszewski, Johannes B. van Goudoever, Martin Post

The third author's name is misspelled. The correct spelling is: Brian P. Kavanagh. The correct citation is: Kroon AA, Wang J, Kavanagh BP, Huang Z, Kuliszewski M, et al. (2011) Prolonged Mechanical Ventilation Induces Cell Cycle Arrest in Newborn Rat Lung. PLoS ONE 6(2): e16910. doi:10.1371/journal.pone.0016910

